# Monocarboxylate transporter expression at the onset of skeletal muscle regeneration

**DOI:** 10.1002/phy2.75

**Published:** 2013-09-10

**Authors:** Tyrone A Washington, Lemuel Brown, Dameon A Smith, Gina Davis, Jamie Baum, Walter Bottje

**Affiliations:** 1 Exercise Muscle Biology Laboratory, University of ArkansasFayetteville, Arkansas, 72701; 2 Human Performance Laboratory, Department of Health, Human Performance and Recreation, University of ArkansasFayetteville, Arkansas, 72701; 3 Food Science Department, University of ArkansasFayetteville, Arkansas, 72701; 4 Poultry Science Department, University of ArkansasFayetteville, Arkansas, 72701

**Keywords:** Bupivacaine injection, CD147 expression, MCT expression, skeletal muscle damage, skeletal muscle regeneration

## Abstract

The onset of skeletal muscle regeneration is characterized by proliferating myoblasts. Proliferating myoblasts have an increased energy demand and lactate exchange across the sarcolemma can be used to address this increased demand. Monocarboxylate transporters (MCTs) are involved in lactate transport across the sarcolemma and are known to be affected by various physiological stimuli. However, MCT expression at the onset of skeletal muscle regeneration has not been determined. The purpose of this study was to determine if skeletal muscle regeneration altered MCT expression in regenerating tibialis anterior (TA) muscle. Male C57/BL6 mice were randomly assigned to either a control (uninjured) or bupivacaine (injured) group. Three days post injection, the TA was extracted for determination of protein and gene expression. A 21% decrease in muscle mass to tibia length (2.4 ± 0.1 mg/mm vs. 1.9 ± 0.2 mg/mm, *P* < 0.02) was observed. IGF-1 and MyoD gene expression increased 5.0-fold (*P* < 0.05) and 3.5-fold (*P* < 0.05), respectively, 3 days post bupivacaine injection. MCT-1 protein was decreased 32% (*P* < 0.03); however, MCT-1 gene expression was not altered. There was no difference in MCT4 protein or gene expression. Lactate dehydrogenase (LDH)-A protein expression increased 71% (*P* < 0.0004). Protein levels of LDH-B and mitochondrial enzyme cytochrome C oxidase subunit decreased 3 days post bupivacaine injection. CD147 and PKC-θ protein increased 64% (*P* < 0.03) and 79% (*P* < 0.02), respectively. MCT1 but not MCT4 expression is altered at the onset of skeletal muscle regeneration possibly in an attempt to regulate lactate uptake and use by skeletal muscle cells.

## Introduction

The ability of skeletal muscle to recover from injury is an important characteristic in all living organisms with impaired skeletal muscle regeneration being associated with numerous pathophysiological conditions such as obesity, diabetes, as well as aging (Hu et al. 2010; Carosio et al. 2011; Tamilarasan et al. 2012). Damage to skeletal muscle is associated with myofiber disruption which induces a local inflammatory response and increased circulating creatine kinase levels (McClung et al. 2007; Washington et al. 2011). In healthy tissues, skeletal muscle recovers rapidly following a damaging stimulus. The skeletal muscle regenerative response requires the coordinated regulation of inflammation, energy metabolism, and myofiber growth (Huard et al. 2002; Ambrosio et al. 2009). Skeletal muscle is postmitotic and therefore skeletal muscle regeneration is dependent on a reserve group of myogenic stem cells called satellite cells (Murphy et al. 2011; Relaix and Zammit 2012). Upon injury, satellite cells are activated and give rise to myoblasts that fuse with the injured myofiber providing additional nuclei increasing their potential for protein synthesis which is necessary for regeneration (Hawke and Garry 2001). Activation, proliferation, and differentiation of myoblasts are modulated by anabolic growth factors such as IGF-1 and the basic helix-loop-helix myogenic regulatory factor (MRFs) family of DNA binding proteins (Rudnicki and Jaenisch 1995; Philippou et al. 2007). The increased expression of IGF-1 and the MRFs, MyoD, and myogenin, coincide with the onset of skeletal muscle growth (Carson et al. 2002; White et al. 2009b).

Lactate is a high-energy molecule that is readily oxidized by skeletal muscle (Brooks 2009). Transport of lactate across the sarcolemma is accomplished by a family of monocarboxylate transporters (MCTs). The MCTs facilitate the 1:1 exchange of lactate and protons across the sarcolemma. These transporters are important in the regulation of intracellular pH, lactate exchange, and cellular metabolism. Regulation of pH is important for optimal cell functioning. During periods of high energy demand, such as cell proliferation, skeletal muscle produces large amounts of lactate that could reduce the internal pH of myoblasts and this would be detrimental to cell viability and function. Therefore, mechanisms aimed at regulating intracellular pH are important. Currently 14 MCTs have been identified; however, MCT1 and MCT4 are believed to be the key MCTs within skeletal muscle (Halestrap and Wilson 2012). MCT1 has a low *K*_m_ (3.5–8.3 mmol/L) for lactate and thus a high affinity for lactate (Halestrap and Price 1999; Halestrap and Wilson 2012). The physiological role of MCT1 appears to be in the uptake of lactate from the circulation (Halestrap and Wilson 2012). Additionally, MCT1 is also found on the inner mitochondrial membrane and functions in the transport of lactate into the mitochondrial matrix (Butz et al. 2004). Conversely, MCT4 appears to favor lactate export (Halestrap and Wilson 2012). CD147 is a glycoprotein that assists MCT1 and MCT4 in folding, stability, membrane expression, and functionality (Kirk et al. 2000). It has been demonstrated that both MCT and CD147 expression can be affected by various physiological stimuli such as skeletal muscle hypertrophy and running (Kitaoka et al. 2011; Thomas et al. 2012). For instance, short-term sprint training is associated with an increase in MCT1 with no change in MCT4 (Bickham et al. 2006). Exercise generally produced greater increases in MCT1 than MCT4 (Thomas et al. 2012). It has also been demonstrated that PGC-1α increases lactate up with concomitant increases in MCT1 but not MCT4 (Benton et al. 2008). CD147 has been well documented to be upregulated during wound healing (Gabison et al. 2005, 2009; Tang et al. 2009).

The early stages of skeletal muscle regeneration are a robust period with the activity of many cells types increased. Proliferating myoblast reach a maximum number between 2 and 3 days following bupivacaine injection (Saito and Nonaka 1994). The onset of skeletal muscle regeneration requires the coordinated regulation of metabolism. Recapitulation of the myogenic program requires energy production for the many events required for successful skeletal muscle regeneration. Genes related to energy metabolism are some of the first that are robustly increased at the onset of skeletal muscle growth (Carson et al. 2002; Washington et al. 2004). The onset of skeletal muscle regeneration is marked by rapid proliferation of myoblast cells (Zhao and Hoffman 2004; Ten Broek et al. 2010). During proliferation the energy requirement of cells increases. For example highly proliferating tumor cells switch from oxidative phosphorylation to glycolysis for energy production (Bensinger and Christofk 2012; Brown et al. 2013). This is referred to as the “Warburg effect”. This is not unique to tumor cells. Upon stimulation, muscle derived cells increased lactate dehydrogenase (LDH) activity while citrate synthase activity decreased (Barani et al. 2003). Furthermore, it has been demonstrated that LDH-A, a glycolytic enzyme, increases during skeletal muscle regeneration in the soleus (Crassous et al. 2009). Taken together this implies that skeletal muscle has the capacity to switch to a glycolytic energy profile to meet the increased energy demands of skeletal muscle regeneration. The onset of skeletal muscle regeneration is a period with robust activity although well defined there is still a considerable void in knowledge about this very active period during skeletal muscle regeneration.

The regulation of energy production and utilization is an important component of skeletal muscle regeneration. The objective of this study was to determine if MCT expression was altered during the onset of skeletal muscle regeneration. We hypothesized that at the onset of skeletal muscle regeneration MCT1 expression would be depressed with little change in MCT4 expression. In addition, we hypothesized that CD147 would be upregulated at the onset of skeletal muscle regeneration.

## Methods

### Animals and housing

Twelve-week old C57BL/6 mice were purchased from Jackson Laboratories. Animals were housed in the University of Arkansas Central Laboratory Animal Facility. Animals were kept on a 12:12-h light-dark cycle, and given access to normal rodent chow and water for the duration of the study. The mice were randomly assigned to one of two groups: (1) uninjured (control; *n* = 4–6) or (2) injured (*n* = 6). All procedures were approved by the University of Arkansas Institutional Animal Care and Use Committee (IACUC).

### Bupivacaine injection

Bupivacaine is a well-established model for damaging skeletal muscle and studying the subsequent skeletal muscle regenerative response (Hall-Craggs 1980; Duguez et al. 2002; Plant et al. 2006; White et al. 2009a). Mice were anesthetized with a subcutaneous injection of a cocktail containing ketamine hydrochloride (45 mg/kg body weight), xylazine (3 mg/kg body weight), and acepromazine (1 mg/kg body weight). Muscle damage was induced by injecting 0.03 mL of 0.75% bupivacaine (Marcaine) in the left and right tibialis anterior (TA). A 25-gauge, 5/8 (0.5 × 16 mm) needle was inserted along the longitudinal axis of the muscle, and the bupivacaine was injected slowly as the needle was withdrawn. Bupivacaine was delivered in an isotonic solution of NaCl. The control group was injected with 0.03 mL of phosphate-buffered saline (PBS).

### Muscle and tibia extraction

Three days post injection, the TA and tibias were extracted. Mice were anesthetized with a subcutaneous injection of a cocktail containing ketamine hydrochloride (90 mg/kg body weight), xylazine (3 mg/kg body weight), and acepromazine (1 mg/kg body weight). The left TA was snap frozen in liquid nitrogen and stored at −80°C for protein and gene expression analysis, and the right TA was cut at the midbelly, mounted in optimum cutting temperature compound (OCT), and then dropped in liquid nitrogen cooled isopentane. Once frozen, samples were stored at −80°C for morphological analysis. After the TA was dissected out, the tibia was removed and measured using a plastic caliper.

### Western blotting

Tissue was homogenized in Mueller Buffer and protein concentration was measured using the Qubit 2.0® (Life Technologies, Grand Island, NY). Muscle homogenate (40 μg) was fractionated into 12% sodium dodecyl sulfate (SDS)-polyacrylamide gels. Gels were transferred overnight to polyvinylidene difluoride (PVDF) membranes. Membranes were Ponceau stained before blotting to verify equal loading of the gels. Membranes were blocked in 5% bovine serum albumin (BSA), in Tris-buffered saline with 0.1% Tween-20 (TBST), for 2 h. Primary antibodies for MCT1 (Santa Cruz, SC-14917), MCT4 (Santa Cruz, SC-14930), LDH-A (Santa Cruz, SC-27230), LDH-B (Abcam, Cambridge, MA, ab85318), COX-IV (Cell Signaling, Boston, MA, 4850P), and PKC-θ (Santa Cruz, SC-212) were diluted 1:2000–1:8000 in 5% BSA or nonfat milk, in TBST, and incubated at room temperature for 1 h or 4°C overnight. Anti-goat secondary antibodies (Santa Cruz, Santa Cruz, CA) were diluted 1:10,000 in 5% BSA or nonfat milk, in TBST, and then incubated at room temperature for 1 h. Enhanced Chemiluminescence (ECL) was performed using Fluorochem M imager (Protein Simple, Santa Clara, CA) to visualize antibody-antigen interaction. Blotting images were quantified by densitometry using AlphaView software (Protein Simple). The Ponceau-stained membranes were digitally scanned, and the 45-kDa actin bands were quantified by densitometry and used as a protein loading correction factor for each lane.

### RNA isolation, cDNA synthesis, and quantitative RT-PCR

RNA was extracted with Trizol reagent (Life Technologies) as previously described (Washington et al. 2004, 2011; White et al. 2009b). Briefly, TA muscles were homogenized in Trizol. Total RNA was isolated, DNase treated and concentration and purity was determined by fluorometry using the Qubit 2.0 (Life Technologies). cDNA was reverse transcribed from 1 μg of total RNA using the Superscript Vilo cDNA synthesis kit (Life Technologies). Real-time polymerase chain reaction (PCR) was performed, and results were analyzed by using the ABI 7300 thermocycler Real-Time detection system (Sequence Detection Systems, model 7300; Applied Biosystems, Foster City, CA). cDNA was amplified in a 25 μL reaction containing appropriate primer pairs and ABI SYBR Green or TaqMan Universal Mastermix (Applied Biosystems). Samples were incubated at 95°C for 4 min, followed by 40 cycles of denaturation, annealing, and extension at 95°C, 55°C, and 72°C, respectively. SYBR Green or TaqMan fluorescence was measured at the end of the extension step each cycle. Fluorescence labeled probes for MyoD (FAM dye), MCT1 (FAM dye), MCT4 (FAM dye), and the ribosomal RNA 18s (VIC dye) were purchased from Applied Biosystems and quantified with TaqMan Universal mastermix. IGF-1 primer sequence was synthesized by Integrated DNA Technologies (IDT) (Caralville, IA) and quantified with SYBR Green mastermix. The primer sequences for IGF-1 were as follows: forward, 5′-TGGATGCTCTTCAGTTCGTG-3′; reverse, 5′-GTCTTGGGCATGTCAGTGTG-3′. Cycle threshold (*C*_t_) was determined, and the Δ*C*_t_ value calculated as the difference between the *C*_t_ value and the 18s *C*_t_ value. Final quantification of gene expression was calculated using the ΔΔCT method *C*_t_ = (Δ*C*_t_ [calibrator]−Δ*C*_t_ [sample]). Relative quantification was then calculated as 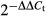. Melt curve analysis was performed at the end of the PCR run to verify that no primer dimers were formed.

### Lactate measurements

Serum and TA muscle lactate concentration was quantified using the L-Lactate Assay kit (Eton Biosciences Inc., Research Triangle Park, NC) per manufacturer's instructions. Duplicate measurements were made for all treatments.

### Percent noncontractile tissue

Percent noncontractile tissue (%NCT) was determined as previously described (White et al. 2009b; Washington et al. 2011). Briefly, approximately eight digital images of H&E-stained sections of TA muscle were analyzed. An 18 × 14 grid overlaid the digital images. Each dot was counted if it was not on a muscle fiber by a blinded investigator. Dots at least 75% in the extracellular matrix (ECM) were counted. Dots that were not clearly distinguishable were omitted from the count.

### Fluorescent microscopy

Serial, transverse cryosections (10 μm thick) of the midbelly region of frozen TA muscles were cut at −20°C using a cryostat (Leica Biosystems, Buffalo Grove, IL). Sections were mounted on histidine coated slides and stored in −80°C. Slides were removed from −80°C and allowed to come to room temperature; any condensation was dried from slides. Sections were rehydrated with PBS, then blocked in blocking solution (PBS + 2% BSA) for 45 min. MCT-1 goat polyclonal antibody (4 μg/mL) (Santa Cruz Biotechnologies) was applied for 1 h at room temperature. As a negative control, the primary antibody was omitted. Alexi Flour 488 anti-goat secondary antibody (4 μg/mL) (Cell Signaling) was incubated for 1 h at room temperature protected from light by a light shield. Prolong Gold with 4′,6-diamidino-2-phenylindole, dihydrochloride (DAPI; Life Technologies) was applied to each slide. Images were taken using Axioskop 2 Plus (Zeiss, Thornwood, NY) at a 40× objective and 10× eyepiece for each muscle section.

### Data analysis

Results are reported as mean ± SE. Data were analyzed using Student's *t*-test. Statistical significance was determined if *P* ≤ 0.05.

## Results

### Muscle mass characteristics, noncontractile tissue, serum, and tissue lactate concentration

**Figure 1:**
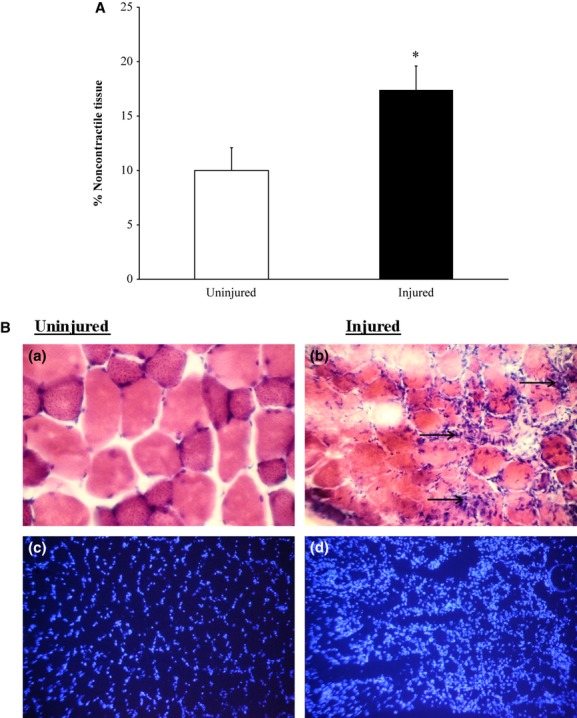
(A) The effect of bupivacaine injection on volume of percentage of noncontractile tissue in the tibialis anterior muscle. (B) Representative H&E staining of muscle cross section of (a) uninjured and (b) injured and representative DAPI staining of (c) uninjured and (d) injured tibialis anterior muscle. The arrows indicate extracellular nuclei. Values are means ± SE. *Difference between uninjured and injured, *P* ≤ 0.05.

At 3 days post bupivacaine injection, TA muscle weight decreased 17% (*P* < 0.05; Table 1) and when TA muscle weight was normalized to tibia length there was a 21% decrease (*P* < 0.05; Table 1). There was no effect of bupivacaine injection on tibia length. The percentage of noncontractile tissue increased 74% (*P* < 0.03; Fig. 1A) 3 days post bupivacaine injection. Lactate concentration in the plasma was not affected by bupivacaine injection; however, there was a trend for a decrease in TA lactate concentration (*P* = 0.1) (Table 1).

**Table 1:** Tibialis anterior muscle weight, tibia length, tibialis anterior muscle weight normalized by tibia length, TA lactate concentration, and plasma lactate concentration 3 days after bupivacaine-induced injury

	Uninjured	Injured
Tibialis anterior (mg)	38.3 ± 1.4	31.9 ± 3.0*
Tibia length (mm)	15.8 ± 0.6	16.8 ± 0.1
Muscle mass/tibia length (mg/mm)	2.4 ± 0.1	1.9 ± 0.2*
TA lactate conc. (μmol/mg protein)	2.0 ± 0.4	1.4 ± 0.3
Plasma lactate conc. (mmol/L)	5.0 ± 0.6	4.2 ± 0.9

Values are means ± SE.

* Significantly different from uninjured animals, *P* < 0.05.

### MRF and anabolic growth factor gene expression

**Figure 2:**
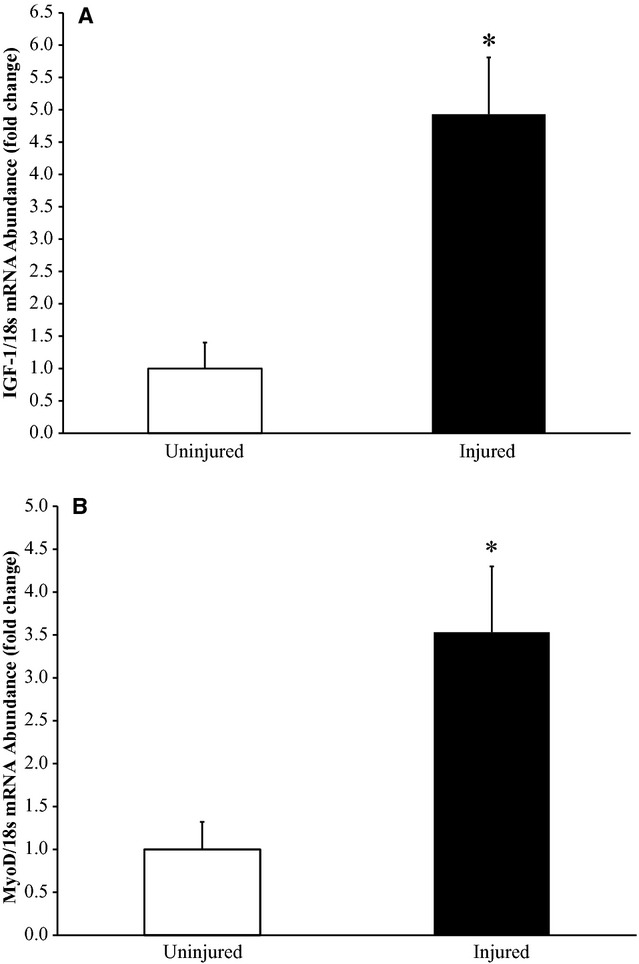
Markers of skeletal muscle regeneration at 3 days of recovery after bupivacaine-induced injury. IGF-1 gene expression shown in (A) and myoD gene expression shown in (B). All data were normalized to the uninjured control. Injured groups were injected with bupivacaine. Uninjured groups were injected with PBS. Values are means ± SE. *Difference between uninjured and injured, *P* ≤ 0.05.

Skeletal muscle regeneration is characterized by a robust inflammatory response. This is marked by infiltrating cells within skeletal muscle. We observed an increase in extracellular nuclei 3 days post bupivacaine injection (Fig. 1B). MyoD and IGF-1 mRNA were quantified as markers of skeletal muscle regeneration. IGF-1 mRNA increased approximately fivefold (*P* < 0.0009) 3 days post bupivacaine injection (Fig. 2A). MyoD mRNA increased approximately fourfold (*P* < 0.008) 3 days post bupivacaine injection (Fig. 2B).

### MCT 1 and 4 expression, MCT 1 localization, and CD147 expression

**Figure 3:**
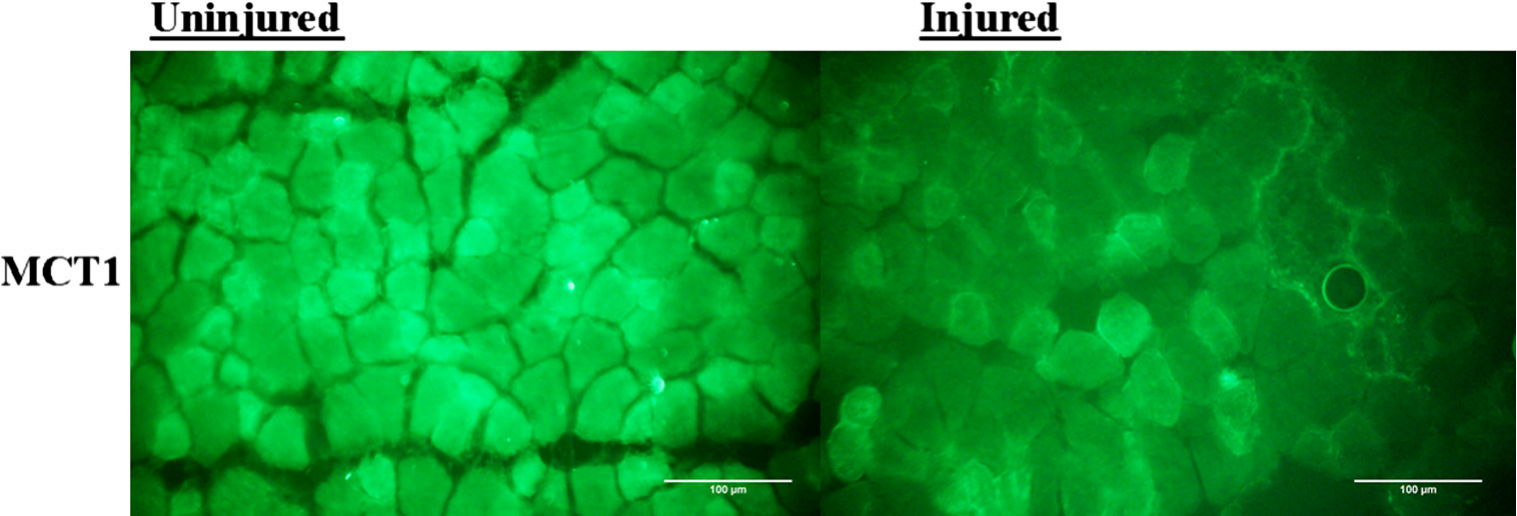
Immunofluorescence staining of MCT1 (200× magnification) 3 days post bupivacaine injection.

**Figure 4:**
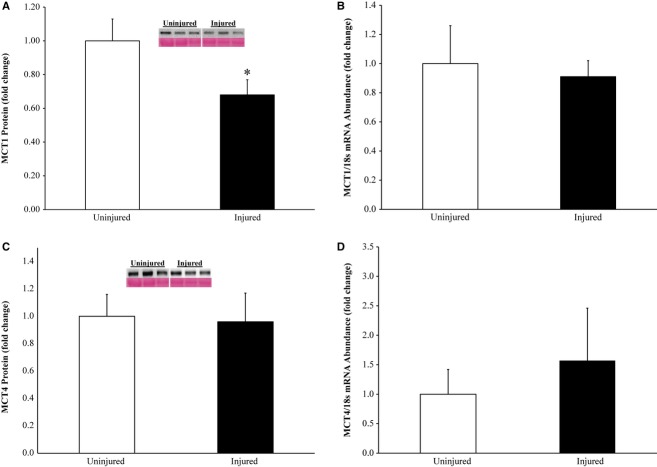
MCT expression 3 days after recovery from bupivacaine-induced injury. (A) The effect of 3 days of recovery from bupivacaine-induced injury on MCT1 protein expression. (B) The effect of 3 days of recovery from bupivacaine-induced injury on MCT1 gene expression. (C) The effect of 3 days of recovery from bupivacaine-induced injury on MCT4 protein expression. (D) The effect of 3 days of recovery from bupivacaine-induced injury on MCT4 gene expression. Inset figure is representative Western blot and Ponceau S stain taken from the same gel and image. Values are means ± SE. *Difference between uninjured and injured, *P* ≤ 0.05.

**Figure 5:**
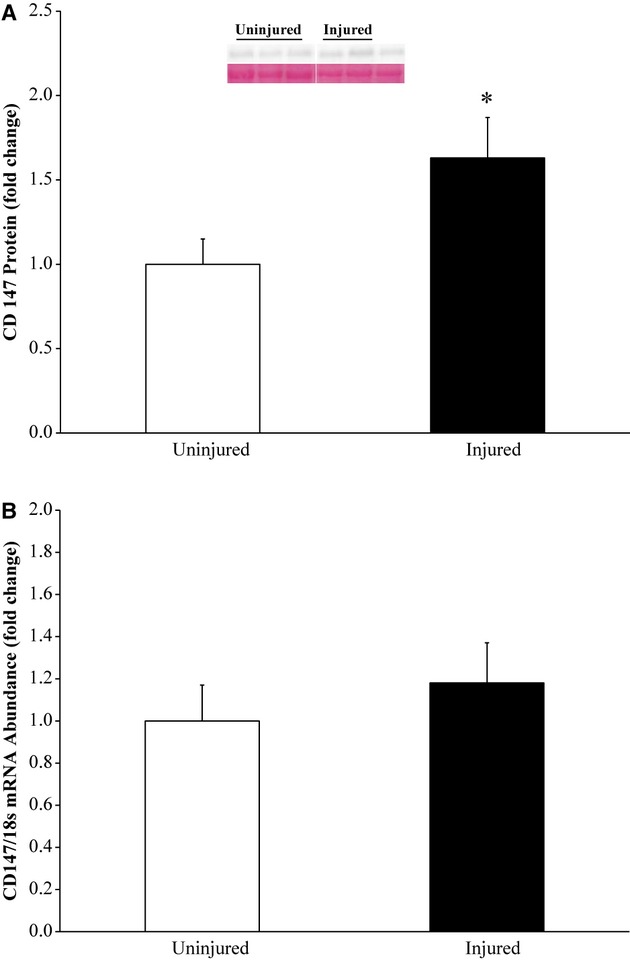
CD147 expression 3 days after recovery from bupivacaine-induced injury. (A) The effect of 3 days of recovery from bupivacaine-induced injury on CD147 protein expression. (B) The effect of 3 days of recovery from bupivacaine-induced injury on CD147 gene expression. Inset figure is representative Western blot and Ponceau S stain taken from the same gel and image. Values are means ± SE. *Difference between uninjured and injured, *P* ≤ 0.05.

MCT1 is a transmembrane protein as seen by localization at the plasma membrane (Fig. 3). MCT1 appears to be decreased at the plasma membrane 3 days post bupivacaine injection (Fig. 3). Corresponding to the decreased MCT1 at the plasma membrane, MCT1 protein expression decreased 32% (*P* < 0.03; Fig. 4A). In contrast, there was no difference in MCT1 mRNA 3 days post bupivacaine injection (Fig. 4B). There were no differences observed in MCT4 protein or gene expression (Fig. 4C and D). There was a 64% increase (*P* < 0.03) in CD147 protein expression 3 days post bupivacaine injection but there was no change in CD147 gene expression (Fig. 5A and B).

### LDH-A, LDH-B, COX-IV, and PKC-θ protein expression

**Figure 6:**
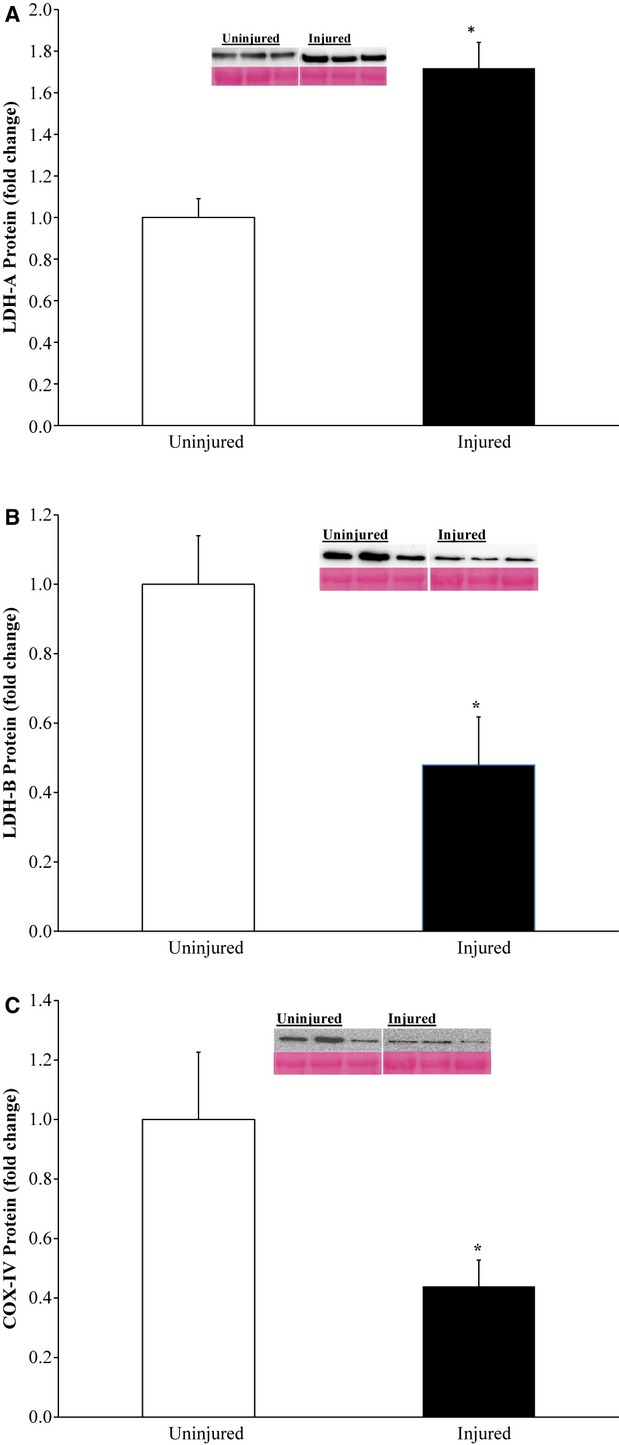
LDH and COX-IV expression 3 days after recovery from bupivacaine-induced injury. (A) The effect of 3 days of recovery from bupivacaine-induced injury on LDH-A protein expression. (B) The effect of 3 days of recovery from bupivacaine-induced injury on LDH-B gene expression. (C) The effect of 3 days of recovery from bupivacaine-induced injury on COX-IV expression. Inset figure is representative Western blot and Ponceau S stain taken from the same gel and image. Values are means ± SE. *Difference between uninjured and injured, *P* ≤ 0.05.

**Figure 7:**
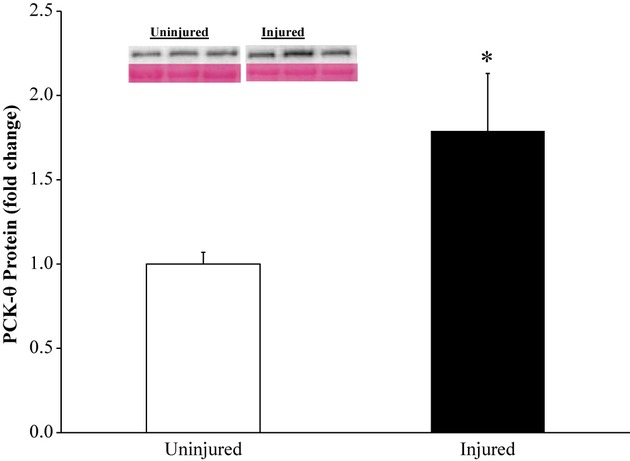
The effect of 3 days of recovery from bupivacaine-induced injury on PKC-θ protein expression. Inset figure is representative Western blot and Ponceau S stain taken from the same gel and image. Values are means ± SE. *Difference between uninjured and injured, *P* ≤ 0.05.

LDH is a tetrameric enzyme that catalyzes the NADH-dependent interconversion of pyruvate to lactate. LDH-A is responsible for the conversion of pyruvate to lactate and LDH-B is responsible for the conversion of lactate to pyruvate. LDH-A increased 71% (*P* < 0.003) 3 days post bupivacaine injection (Fig. 6A). LDH-B decreased 53% (*P* < 0.05) 3 days post bupivacaine injection (Fig. 6B). Cytochrome C oxidase subunit IV (COX-IV) decreased 57% (*P* < 0.05) 3 days post bupivacaine injection (Fig. 6C). PKC-θ protein expression increased 79% (*P* < 0.02) 3 days post bupivacaine injection (Fig. 7).

## Discussion

The primary objective of this study was to examine MCT expression at the onset of skeletal muscle regeneration. The results from this study extend our understanding of MCT expression within skeletal muscle at onset skeletal muscle regeneration. To our knowledge this is the first to report the novel finding that the expression levels of certain members of the MCT family are altered at the onset of skeletal muscle regeneration. Specifically, our data demonstrated a downregulation of MCT1 protein expression whereas there was no change in MCT4 protein expression after treatment with bupivacaine. In addition, our data also demonstrate the novel finding that CD147 is upregulated at the onset of skeletal muscle regeneration.

MCT1 protein levels decreased at the onset of skeletal muscle regeneration. MCT1 is associated with uptake or efflux of monocarboxylates (i.e., lactate) through the sarcolemma that depends on the metabolic need of the cells. During skeletal muscle regeneration, for example, the energy demand by the proliferating myoblasts is high (Barani et al. 2003; Crassous et al. 2009). When myoblasts were stimulated with serum for 7 days there was a marked upregulation of LDH activity and a concomitant downregulation of citrate synthase activity (Barani et al. 2003). Thus, proliferating myoblasts seem to have an increased dependence on glycolytic metabolism. Additionally, bupivacaine injection decreases citrate synthase activity 80% 3 days post injection and is associated with a fourfold decrease in mitochondrial protein yield (Duguez et al. 2002). Our data are consistent with skeletal muscle switching to a more anaerobic energy profile during a time when myoblasts a robustly proliferating. We demonstrated an increase in LDH-A protein levels 3 days following bupivacaine injection. We examined enzymes related to oxidative metabolism. We measured LDH-B which is the enzyme responsible for the conversion of lactate to pyruvate and COX-IV, which is a mitochondrial DNA encoded protein and corresponds well to mitochondrial biogenesis (Laye et al. 2009; Geng et al. 2010). We showed a decrease in LDH-B and COX-IV protein levels. This coincides with maximal satellite cell/myoblast proliferation (Goetsch et al. 2003).

Increased flux through glycolysis necessitates a need for pH control. Many processes within skeletal muscle are influenced by pH and therefore it is important for pH to be tightly regulated for the cell to function optimally. During fatiguing contractions the internal pH within skeletal muscle can decrease to ∼6.5 due to lactate accumulation (Juel 1996). The ability to regulate lactate/H^+^ entry and exit from skeletal muscle is extremely important for proper muscle functioning. Our data provide evidence for another mechanism that skeletal muscle may use to prevent major decrements in intracellular pH. The decrease in MCT1 observed in this study could point to the cell regulating the entry of lactate and thus H^+^ from the circulation. We show that lactate concentration in the plasma was not different between the uninjured and injured group. There was a trend for a decrease in lactate concentration in the TA muscle. This might suggest that the lactate is being utilized by the cell for energy.

CD147, also known an ECM proteinase inducer (EMMPRIN), is best known for its role in inducing matrix metalloproteinases (MMPs) expression (Iacono et al. 2007). CD147 coimmunoprecipitates with MCT1 and MCT4 (Kirk et al. 2000). It has been suggested that CD147 is needed for correct localization of MCTs to the plasma membrane (Kirk et al. 2000). With CD147 increasing and MCT1 decreasing during skeletal muscle regeneration, another role besides aiding in MCT docking for CD147 seems likely. Muscle fibers are surrounded by the ECM and ECM regulation is critical for an optimal growth response in skeletal muscle (Goetsch et al. 2003; White et al. 2009b). The ECM acts as a barrier for diffusion and also provides mechanical strength and elasticity. MMPs are involved in the degradation of the ECM which allows myoblasts cells to proliferate and migrate within skeletal muscle tissue (Torrente et al. 2000; Nishimura et al. 2008). Cytokines and growth factors are sequestered within the ECM and their release is critical during skeletal muscle regeneration (Martin 1997; Reddi 1998). MMPs are involved in the liberation of these factors. Our data provide additional evidence to support the role of ECM remodeling during skeletal muscle regeneration. We are the first to show an upregulation of CD147 at the onset of skeletal muscle regeneration. This coincides with increased MMP2 and MMP9 levels that have been demonstrated in numerous studies (Goetsch et al. 2003; Zimowska et al. 2008).

The injection of bupivacaine into skeletal muscle is associated with extensive myofiber necrosis and loss of muscle mass (Beitzel et al. 2004; White et al. 2009a). The subsequent regenerative response requires the myogenesis particularly during the initial phase of skeletal muscle regeneration. The initial regenerative response involves the induction of inflammatory genes that is characterized by infiltration of neutrophils within the first 24 h followed by macrophages (McClung et al. 2007; Segawa et al. 2008; Smith et al. 2008). These cells are involved in the removal of necrotic tissue. Skeletal muscle regeneration is dependent on satellite cells (Relaix and Zammit 2012) and their activation and proliferation peak 2–3 days post bupivacaine injection (Marsh et al. 1997; McLoon et al. 1998; Hawke and Garry 2001). IGF-1 is a potent mitogen that has been demonstrated to induce myogenesis (Adams and Haddad 1996). IGF-1 mRNA is induced at the onset of skeletal muscle growth (Adams and Haddad 1996; White et al. 2009b). We have previously demonstrated that when skeletal muscle regeneration is hindered it is also associated with depressed IGF-1 gene expression and associated signaling (Washington et al. 2011). Importantly for this study, the induction of IGF-1 confirms the efficacy of the injury/regeneration model used.

MCT1 is ubiquitously expressed in skeletal muscle. MCT1 has been reported to be responsible for lactic acid uptake by L6 skeletal muscle cells (Kobayashi et al. 2004). The intracellular regulation of MCT1 is important for understanding the physiological homeostasis within skeletal muscle. It has been demonstrated that MCT1 can be regulated by PKC (Narumi et al. 2010). Phorbol 12-myristate 13-acetate, a PKC activator, was shown to increase lactic acid uptake in rhabdomyosarcoma cells as well as to increase MCT1 protein and mRNA levels (Narumi et al. 2010). In addition, the addition of bisindolylmaleimide, a PKC inhibitor, abolished these effects (Narumi et al. 2010). Our findings do not support the role of PKC-θ in regulating MCT1 during the onset of skeletal muscle regeneration. Our data demonstrated a decrease in MCT1 protein expression. However, there was a significant increase in PKC-θ protein expression. There are multiple isoforms of PKC and it is possible that MCT1 is being regulated by another isoform of PKC at the onset of skeletal muscle regeneration. The underlying mechanism behind MCT1 expression during skeletal muscle regeneration warrants further study.

In summary, the onset of skeletal muscle regeneration is associated with altered expression of MCT1. In addition, CD147 was upregulated at the onset of skeletal muscle regeneration. Proliferating skeletal muscle cells are relying increasingly on anaerobic metabolism to meet their energy demands. These data provide a way that skeletal muscle can increase energy production to match the increased energy demands during the onset of skeletal muscle regeneration via anaerobic metabolism while decreasing uptake of exogenous lactate possibly to regulate pH.

## References

[b1] Adams GR, Haddad F (1996). The relationships among IGF-1, DNA content, and protein accumulation during skeletal muscle hypertrophy. J. Appl. Physiol.

[b2] Ambrosio F, Kadi F, Lexell J, Fitzgerald GK, Boninger ML, Huard J (2009). The effect of muscle loading on skeletal muscle regenerative potential: an update of current research findings relating to aging and neuromuscular pathology. Am. J. Phys. Med. Rehabil.

[b3] Barani AE, Durieux AC, Sabido O, Freyssenet D (2003). Age-related changes in the mitotic and metabolic characteristics of muscle-derived cells. J. Appl. Physiol.

[b4] Beitzel F, Gregorevic P, Ryall JG, Plant DR, Sillence MN, Lynch GS (2004). Beta2-adrenoceptor agonist fenoterol enhances functional repair of regenerating rat skeletal muscle after injury. J. Appl. Physiol.

[b5] Bensinger SJ, Christofk HR (2012). New aspects of the Warburg effect in cancer cell biology. Semin. Cell Dev. Biol.

[b6] Benton CR, Yoshida Y, Lally J, Han XX, Hatta H, Bonen A (2008). PGC-1alpha increases skeletal muscle lactate uptake by increasing the expression of MCT1 but not MCT2 or MCT4. Physiol. Genomics.

[b7] Bickham DC, Bentley DJ, Cameron-Smith PF, Le Rossignol D (2006). The effects of short-term sprint training on MCT expression in moderately endurance-trained runners. Eur. J. Appl. Physiol.

[b8] Brooks GA (2009). Cell-cell and intracellular lactate shuttles. J. Physiol.

[b9] Brown NJ, Higham SE, Perunovic B, Arafa M, Balasubramanian S, Rehman I (2013). Lactate dehydrogenase-B is silenced by promoter methylation in a high frequency of human breast cancers. PLoS One.

[b10] Butz CE, McClelland GB, Brooks GA (2004). MCT1 confirmed in rat striated muscle mitochondria. J. Appl. Physiol.

[b11] Carosio S, Berardinelli MG, Aucello M, Musaro A (2011). Impact of ageing on muscle cell regeneration. Ageing Res. Rev.

[b12] Carson JA, Nettleton D, Reecy JM (2002). Differential gene expression in the rat soleus muscle during early work overload-induced hypertrophy. FASEB J.

[b13] Crassous B, Richard-Bulteau H, Deldicque L, Serrurier B, Pasdeloup M, Francaux M (2009). Lack of effects of creatine on the regeneration of soleus muscle after injury in rats. Med. Sci. Sports Exerc.

[b14] Duguez S, Feasson L, Denis C, Freyssenet D (2002). Mitochondrial biogenesis during skeletal muscle regeneration. Am. J. Physiol. Endocrinol. Metab.

[b15] Gabison EE, Hoang-Xuan T, Mauviel A, Menashi S (2005). EMMPRIN/CD147, an MMP modulator in cancer, development and tissue repair. Biochimie.

[b16] Gabison EE, Huet E, Baudouin C, Menashi S (2009). Direct epithelial-stromal interaction in corneal wound healing: role of EMMPRIN/CD147 in MMPs induction and beyond. Prog. Retin. Eye Res.

[b17] Geng T, Li P, Okutsu M, Yin X, Kwek J, Zhang M (2010). PGC-1alpha plays a functional role in exercise-induced mitochondrial biogenesis and angiogenesis but not fiber-type transformation in mouse skeletal muscle. Am. J. Physiol. Cell Physiol.

[b18] Goetsch SC, Hawke TJ, Gallardo TD, Richardson JA, Garry DJ (2003). Transcriptional profiling and regulation of the extracellular matrix during muscle regeneration. Physiol. Genomics.

[b19] Halestrap AP, Price NT (1999). The proton-linked monocarboxylate transporter (MCT) family: structure, function and regulation. Biochem. J.

[b20] Halestrap AP, Wilson MC (2012). The monocarboxylate transporter family – role and regulation. IUBMB Life.

[b21] Hall-Craggs EC (1980). Early ultrastructural changes in skeletal muscle exposed to the local anaesthetic bupivacaine (Marcaine). Br. J. Exp. Pathol.

[b22] Hawke TJ, Garry DJ (2001). Myogenic satellite cells: physiology to molecular biology. J. Appl. Physiol.

[b23] Hu Z, Wang H, Lee IH, Modi S, Wang X, Du J (2010). PTEN inhibition improves muscle regeneration in mice fed a high-fat diet. Diabetes.

[b24] Huard J, Li Y, Fu FH (2002). Muscle injuries and repair: current trends in research. J. Bone Joint Surg. Am.

[b25] Iacono KT, Brown AL, Greene MI, Saouaf SJ (2007). CD147 immunoglobulin superfamily receptor function and role in pathology. Exp. Mol. Pathol.

[b26] Juel C (1996). Lactate/proton co-transport in skeletal muscle: regulation and importance for pH homeostasis. Acta Physiol. Scand.

[b27] Kirk P, Wilson MC, Heddle C, Brown MH, Barclay AN, Halestrap AP (2000). CD147 is tightly associated with lactate transporters MCT1 and MCT4 and facilitates their cell surface expression. EMBO J.

[b28] Kitaoka Y, Machida M, Takemasa T, Hatta H (2011). Expression of monocarboxylate transporter (MCT) 1 and MCT4 in overloaded mice plantaris muscle. J. Physiol. Sci.

[b29] Kobayashi M, Itagaki S, Hirano T, Iseki K (2004). Mechanism of L-lactic acid transport in L6 skeletal muscle cells. Drug Metab. Pharmacokinet.

[b30] Laye MJ, Rector RS, Warner SO, Naples SP, Perretta AL, Uptergrove GM (2009). Changes in visceral adipose tissue mitochondrial content with type 2 diabetes and daily voluntary wheel running in OLETF rats. J. Physiol.

[b31] Marsh DR, Criswell DS, Carson JA, Booth FW (1997). Myogenic regulatory factors during regeneration of skeletal muscle in young, adult, and old rats. J. Appl. Physiol.

[b32] Martin P (1997). Wound healing – aiming for perfect skin regeneration. Science.

[b33] McClung JM, Davis JM, Carson JA (2007). Ovarian hormone status and skeletal muscle inflammation during recovery from disuse in rats. Exp. Physiol.

[b34] McLoon LK, Nguyen LT, Wirtschafter J (1998). Time course of the regenerative response in bupivacaine injured orbicularis oculi muscle. Cell Tissue Res.

[b35] Murphy MM, Lawson JA, Mathew SJ, Hutcheson DA, Kardon G (2011). Satellite cells, connective tissue fibroblasts and their interactions are crucial for muscle regeneration. Development.

[b36] Narumi K, Furugen A, Kobayashi M, Otake S, Itagaki S, Iseki K (2010). Regulation of monocarboxylate transporter 1 in skeletal muscle cells by intracellular signaling pathways. Biol. Pharm. Bull.

[b37] Nishimura T, Nakamura K, Kishioka Y, Kato-Mori Y, Wakamatsu J, Hattori A (2008). Inhibition of matrix metalloproteinases suppresses the migration of skeletal muscle cells. J. Muscle Res. Cell Motil.

[b38] Philippou A, Halapas A, Maridaki M, Koutsilieris M (2007). Type I insulin-like growth factor receptor signaling in skeletal muscle regeneration and hypertrophy. J. Musculoskelet. Neuronal Interact.

[b39] Plant DR, Colarossi FE, Lynch GS (2006). Notexin causes greater myotoxic damage and slower functional repair in mouse skeletal muscles than bupivacaine. Muscle Nerve.

[b40] Reddi AH (1998). Role of morphogenetic proteins in skeletal tissue engineering and regeneration. Nat. Biotechnol.

[b41] Relaix F, Zammit PS (2012). Satellite cells are essential for skeletal muscle regeneration: the cell on the edge returns centre stage. Development.

[b42] Rudnicki MA, Jaenisch R (1995). The MyoD family of transcription factors and skeletal myogenesis. Bioessays.

[b43] Saito Y, Nonaka I (1994). Initiation of satellite cell replication in bupivacaine-induced myonecrosis. Acta Neuropathol.

[b44] Segawa M, Fukada S, Yamamoto Y, Yahagi H, Kanematsu M, Sato M (2008). Suppression of macrophage functions impairs skeletal muscle regeneration with severe fibrosis. Exp. Cell Res.

[b45] Smith C, Kruger MJ, Smith RM, Myburgh KH (2008). The inflammatory response to skeletal muscle injury: illuminating complexities. Sports Med.

[b46] Tamilarasan KP, Temmel H, Das SK, Al Zoughbi W, Schauer S, Vesely PW (2012). Skeletal muscle damage and impaired regeneration due to LPL-mediated lipotoxicity. Cell Death Dis.

[b47] Tang Z, Yang L, Zhang J, Xue R, Wang Y, Chen PC (2009). Coordinated expression of MMPs and TIMPs in rat knee intra-articular tissues after ACL injury. Connect. Tissue Res.

[b48] Ten Broek RW, Grefte S, Von den Hoff JW (2010). Regulatory factors and cell populations involved in skeletal muscle regeneration. J. Cell. Physiol.

[b49] Thomas C, Bishop DJ, Lambert K, Mercier J, Brooks GA (2012). Effects of acute and chronic exercise on sarcolemmal MCT1 and MCT4 contents in human skeletal muscles: current status. Am. J. Physiol. Regul. Integr. Comp. Physiol.

[b50] Torrente Y, Caron E, El Fahime NJ, Bresolin N, Tremblay JP (2000). Intramuscular migration of myoblasts transplanted after muscle pretreatment with metalloproteinases. Cell Transplant.

[b51] Washington TA, Reecy JM, Thompson RW, Lowe LL, McClung JM, Carson JA (2004). Lactate dehydrogenase expression at the onset of altered loading in rat soleus muscle. J. Appl. Physiol.

[b52] Washington TA, White JP, Davis JM, Wilson LB, Lowe LL, Sato S (2011). Skeletal muscle mass recovery from atrophy in IL-6 knockout mice. Acta Physiol. (Oxf).

[b53] White JP, Baltgalvis KA, Sato S, Wilson LB, Carson JA (2009a). Effect of nandrolone decanoate administration on recovery from bupivacaine-induced muscle injury. J. Appl. Physiol.

[b54] White JP, Reecy JM, Washington TA, Sato S, Le ME, Davis JM (2009b). Overload-induced skeletal muscle extracellular matrix remodelling and myofibre growth in mice lacking IL-6. Acta Physiol. (Oxf).

[b55] Zhao P, Hoffman EP (2004). Embryonic myogenesis pathways in muscle regeneration. Dev. Dyn.

[b56] Zimowska M, Brzoska E, Swierczynska M, Streminska W, Moraczewski J (2008). Distinct patterns of MMP-9 and MMP-2 activity in slow and fast twitch skeletal muscle regeneration in vivo. Int. J. Dev. Biol.

